# Laboratory parameters in lean NAFLD: comparison of subjects with lean NAFLD with obese subjects without hepatic steatosis

**DOI:** 10.1186/s13104-018-3212-1

**Published:** 2018-02-06

**Authors:** Philipp Bernhardt, Wolfgang Kratzer, Julian Schmidberger, Tilmann Graeter, Beate Gruener, for the EMIL Study Group, G. Adler, A. Armsen, H.-M. Banzhaf, M. Bauerdick, P. Bernhardt, U. Bertling, B. O. Boehm, B. O. Brandner, S. O. Brockmann, M. Deckert, C. Dingler, S. Eggink, M. Fuchs, W. Gaus, H. Goussis, A. Gruenert, M. M. Haenle, W. Hampl, C. Haug, B. Hay, L. Heitz, M.-L. Huetter, N. Iftikhar, A. Imhof, T. Kaltenbach, P. Kern, P. Kimmig, A. Kirch, D. Klass, W. Koenig, W. Kratzer, M. Kron, B. Manfras, K. Meitinger, T. Mertens, R. Oehme, G. Pfaff, I. Piechotowski, S. Reuter, T. Romig, A. F. A. von Schmiesing, S. Stanosek, G. Steinbach, M. Tourbier, A. Voegtle, T. Walcher, S. Wolff

**Affiliations:** 1 Department of Internal Medicine I, Ulm University Hospital, Albert-Einstein-Allee 23, 89081 Ulm, Germany; 2 Department of Diagnostic and Interventional Radiology, Ulm University Hospital, Albert-Einstein-Allee 23, 89081 Ulm, Germany; 3 Department of Internal Medicine III, Ulm University Hospital, Albert-Einstein-Allee 23, 89081 Ulm, Germany

**Keywords:** Ferritin, Haemoglobin, Haematocrit, Mean corpuscular haemoglobin concentration, Waist-to-hip ratio, lean NAFLD

## Abstract

**Objective:**

Search for meaningful laboratory and anthropometric parameters in lean non-alcoholic fatty liver disease (lean NAFLD) in the general population. Out of 2445 subjects in a random population sample, we compared those who had a body mass index (BMI) < 25 and a fatty liver [lean NAFLD (LN), n = 5] with obese subjects who had a BMI > 30 but no fatty liver [non-NAFLD (NN), n = 27] in a follow-up examination. Ultrasonic, anthropometric and laboratory parameters were collected.

**Results:**

There were significant differences (p < 0.05) between the LN and the NN groups with respect to serum ferritin (199.2 ± 72.1 LN vs 106.0 ± 89.6 NN), haemoglobin (14.9 ± 0.8 LN vs 13.5 ± 1.2 NN), haematocrit (0.438 ± 0.019 LN vs 0.407 ± 0.035 NN) and Mean corpuscular haemoglobin concentration (34 ± 0.6 LN vs 33.2 ± 0.8 NN). Significantly lower values of soluble transferrin receptor were measured in the LN group (2.8 ± 0.4 LN vs 3.8 ± 1.5 NN). In both groups, the measured HOMA-IR index (homeostatic model assessment of insulin resistance index) (2.3; normal range ≤ 1) was abnormal. Mean cholesterol (6.2 ± 1.4 LN and 5.6 ± 1.1 NN) and low-density lipoprotein levels (3.8 ± 1.0 LN 3.4 ± 0.9 NN) were above the upper limit of normal in both groups, as was the mean triglycerides level in the LN group (2.6 ± 2.0). In summary, there are differences in parameters of iron and fat metabolism between subjects with LN and overweight subjects without fatty liver infiltration.

**Electronic supplementary material:**

The online version of this article (10.1186/s13104-018-3212-1) contains supplementary material, which is available to authorized users.

## Introduction

Non-alcoholic fatty liver disease (NAFLD) is one of the most common causes of chronic liver failure, both in western industrialised nations and in developing countries.

The assumption that the body mass index (BMI) is a good clinical prognostic marker for identifying patients at risk and initiating therapy has to be questioned, as NAFLD can also occur in people with a normal BMI (lean NAFLD).

In past years, the waist-to-hip ratio (WHR) has been considered one of the best markers for estimating the prognosis [1]. It has yet to be determined whether this is also the case for slim people. Previous studies on lean NAFLD have been carried out predominantly in regions of Asia, which considerably limits the extent to which we can extrapolate them to European or North American populations. Comparison of the results is also hampered by the different definitions of obesity [2–4].

Based on a random sample of the general population taken in 2002, we carried out a study to determine the biomarker profiles of subjects with lean NAFLD and obese subjects without fatty liver disease.

Subjects were selected from the population included in the 2002 *Echinococcus multilocularis* and internal diseases in Leutkirch (EMIL I) study, as that investigation had identified subjects with lean NAFLD [5]. These subjects, still manifesting the condition 11 years later, had had hepatic steatosis with a normal weight for a long period. The same applied to the overweight subjects without fatty liver disease.

Many comparable studies had pre-selected populations, as they enrolled patients having medical check-ups. Recruitment from a random sample in the Allgäu region [of Germany] may allow a better use of the results with respect to the general population.

## Main text

### Materials and methods

**Fig. 1:**
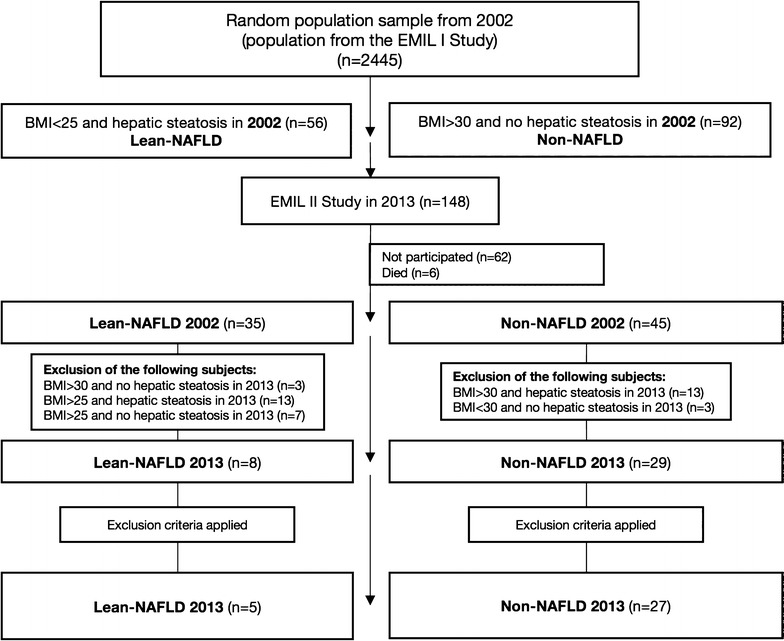
Composition of the subject population for the EMIL IIa study in 2013, showing the recruitment of subjects out of the EMIL I study population from 2002. EMIL, *Echinococcus multilocularis* and other medical conditions in Leutkirch; lean NAFLD, non-alcoholic fatty liver disease in slim people (BMI < 25); non-NAFLD, subjects with a high BMI (> 30) and no hepatic steatosis

The study population comprised 148 persons (age range 20–75 years) from the Leutkirch metropolitan district in southern Germany, recruited from the EMIL-I study population. After analysing the data from 2002, we defined two subgroups of interest (Fig. 1):

- Subjects with a BMI < 25 and demonstrable hepatic steatosis (n = 56):
**lean NAFLD group (LN)**
- Subjects with a BMI > 30 without any demonstrable hepatic steatosis (n = 92):
**non-NAFLD group (NN)**

Out of the 148 subjects, 80 (54.1%) took part in the follow-up study in 2013. Six of the population of 2002 had died and 62 did not participate. Of the 80 participating subjects, 43.6% were in the lean NAFLD group and 56.3% in the non-NAFLD group (Fig. 1).

### Measurement tools

The standardised interview was the same as in the previous study (information about the subjects themselves, their leisure activities, past medical history, eating habits, tobacco use, alcohol consumption and drug use, as well as whether the women were taking contraceptives) [5].

Height, hip and waist measurements were made according to the World Health Organisation (WHO) guidelines using a height scale/tape measure [6].

The ultrasound examination of the liver was examined primarily for fatty infiltration (hepatic steatosis). The size of the liver was determined from the maximum craniocaudal length in the midclavicular line. We used the criteria comparing the liver and kidney parenchyma as defined by Saverymuttu, Hamaguchi and Charatcharoenwitthaya to diagnose hepatic steatosis, taking the posterior ultrasound wave attenuation, the imaging of the diaphragm and assessment of the hepatic vessels into consideration [7]. Patients were then divided into groups: no fatty changes, grade I, grade II, and grade III hepatic steatosis. A Philips IU22 ultrasound scanner (Philips GmbH, Healthcare Division, Hamburg, Germany) was used for all the examinations.

The homeostasis model assessment of insulin resistance (HOMA-IR index) was used in the study as a measure of the insulin resistance. Subjects were asked to fast for 12 h beforehand. A HOMA-IR index of ≤ 1 is considered normal, while an index > 2.0 can be interpreted as evidence of insulin resistance [8]. Different cut-off values were determined for men and women, as well as for metabolic syndrome and NAFLD [9].

The ratio of proinsulin to insulin was used as a measure of possible beta cell dysfunction [10].

All the blood samples were taken and analysed in 2013. The local Institute of Clinical Chemistry, which has DIN EN ISO 15189 accreditation, carried out all the tests on the blood samples.

### Exclusion criteria

The exclusion criteria were pre-existing liver disease, positive hepatitis serology, excessive alcohol consumption (> 20 g/day in women, > 40 g/day in men), raised transferrin saturation and thyroid dysfunction [abnormal triiodothyronine (T3), thyroxine (T4), or thyroid-stimulating hormone (TSH) levels].

### Statistical analysis

Calculations were made using the SAS program (Version 9.2; SAS Institute Inc., Cary, NC, USA). Descriptive methods were used for the frequency of severity and distribution of the target and influencing parameters. Relative and absolute frequencies were determined for qualitative or categorical characteristics. The mean value and standard deviation, as well as the median with minimum, maximum and first and third quartiles were calculated for all quantitative or continuous variables. Some of these are shown in box plots.

We used the Wilcoxon rank sum test to determine differences in continuous variables between the two groups with a normal distribution. A p-value of α = 5% was taken to be significant. The p-value was rounded off and presented according to Bailar et al. [11].

## Results

From the initial population (80 subjects) we were able to enrol 32 subjects (22 women, 10 men) in the present study. The mean BMI was 23.8 (23.0–24.7) in the LN group and 34.0 (27.5–46.5) in the NN group (Table 1). The waist-to-hip ratio (WHR) was 0.915 in the LN group and 0.854 in the NN group. The mean liver size was 13.2 cm (11.9–14.7) in the LN group and 13.4 cm (11.0–15.7) in the NN group (Table 1).

**Table 1:** Blood tests results of subjects in the 2013 EMIL IIa study

	BMI < 25 with fatty liver (n = 5)				BMI ≥ 30 without fatty liver (n = 27)				p-value
	Mean	SD	Min	Max	Mean	SD	Min	Max	
ALT (U/l)	26.0	7.1	19.0	37.0	23.5	7.8	12.0	47.0	0.3627
AST (U/l)	26.4	3.0	23.0	30.0	23.1	4.6	14.0	33.0	0.1235
GGT (U/l)	33.0	14.5	22.0	58.0	38.3	52.3	9.0	279.0	0.2314
AP (U/l)	69.2	18.4	42.0	87.0	71.1	19.4	41.0	133.0	0.9793
Cortisol (µg/dl)	15.5	3.2	10.9	19.4	12.7	6.3	0.6	31.9	0.1880
SHBG (nmol/l)	52.3	22.9	19.8	71.9	60.9	24.4	12.3	121.9	0.5368
Proinsulin (pmol/l)	2.6	2.2	0.7	4.4	2.9	1.9	0.4	7.9	0.6868
Insulin (mU/l)	9.0	3.7	5.8	15.1	9.6	5.4	2.7	27.6	0.9144
Proinsulin/insulin-ratio	0.3	0.3	0.1	0.4	0.3	0.2	0.1	0.9	0.7350
Glucose (mg/dl)	105.0	15.1	87.0	128.0	98.8	11.9	81.0	135.0	0.2749
Fibrinogen (g/l)	2.9	0.7	2.2	4.0	3.6	0.7	2.4	4.9	0.0636
Caeruloplasmin (g/l)	0.22	0.04	0.17	0.27	0.25	0.03	0.17	0.31	0.0682
Serum ferritin (µg/dl)	199.2	72.1	133.0	318.0	106.0	89.6	8.0	408.0	*0.0224*
Albumin (g/l)	47.0	2.4	45.0	51.0	45.1	2.9	40.0	52.0	0.1359
T3 (nmol/l)	1.8	0.2	1.5	2.1	1.6	0.3	0.8	2.1	0.2065
T4 (nmol/l)	99.1	15.8	72.7	112.5	106.0	23.1	52.5	147.0	0.4361
TSH (mIU/l)	1.3	0.6	0.7	2.1	1.6	1.0	0.4	5.3	0.7270
AntiTPO (IU/ml)	12.6	4.4	7.0	18.0	40.5	79.5	5.0	321.0	0.7465
Cholesterol (mmol/l)	6.2	1.4	4.8	8.4	5.6	1.1	3.2	7.9	0.4992
LDL (mmol/l)	3.8	1.0	2.8	5.4	3.4	0.9	1.4	5.2	0.4660
HDL (mmol/l)	1.5	0.6	0.9	2.5	1.7	0.3	1.2	2.2	0.2733
Triglycerides (mmol/l)	2.6	2.0	1.1	5.7	1.2	0.5	0.3	2.1	0.1244
Iron (µmol/l)	18.1	2.9	14.4	22.0	17.5	9.1	7.5	50.2	0.2325
Transferrin (g/l)	2.7	0.3	2.5	3.2	2.6	0.5	1.9	3.9	0.2974
STFR (mg/l)	2.8	0.4	2.4	3.5	3.8	1.5	2.0	8.9	*0.0485*
HOMA-IR	2.3	0.7	1.6	3.2	2.3	1.4	0.6	7.4	0.7677
Red blood cells (× 10^12^/l)	4.8	0.4	4.4	5.4	4.4	0.4	2.9	5.0	0.0931
White blood cells (× 10^9^/l)	5.3	1.2	3.7	7.1	6.2	1.5	4.3	9.2	0.2263
Haemoglobin (g/dl)	14.9	0.8	14.2	16.0	13.5	1.2	9.7	15.7	*0.0078*
Haematocrit (l/l)	0.44	0.02	0.42	0.47	0.41	0.04	0.29	0.47	*0.0227*
MCH (pg/pg)	31.4	1.6	29.5	32.8	30.5	1.5	26.5	33.9	0.3467
MCHC (g/dl)	34.0	0.6	33.0	34.6	33.2	0.8	31.4	34.4	*0.0293*
MCV(fl/fl)	92.6	4.7	86.6	98.3	91.8	3.7	81.9	100.9	0.7676
Platelets (× 10^9^/l)	180.2	59.0	136.0	283.0	214.0	51.0	95.0	332.0	0.0719
HbA1c (%)	5.8	0.7	5.1	7.0	5.7	0.5	5.0	7.4	0.9705
MPV (fl/fl)	10.0	1.6	8.2	11.9	9.2	1.0	7.3	11.2	0.3332

Comparison of the lean NAFLD (BMI < 25 + fatty liver) and non-NAFLD (BMI > 30 and no fatty liver) groups [EMIL, *Echinococcus multilocularis* and other medical conditions in Leutkirch; SD, standard deviation]

Italic values indicate significance of p values (p < 0.05)

**Fig. 2:**
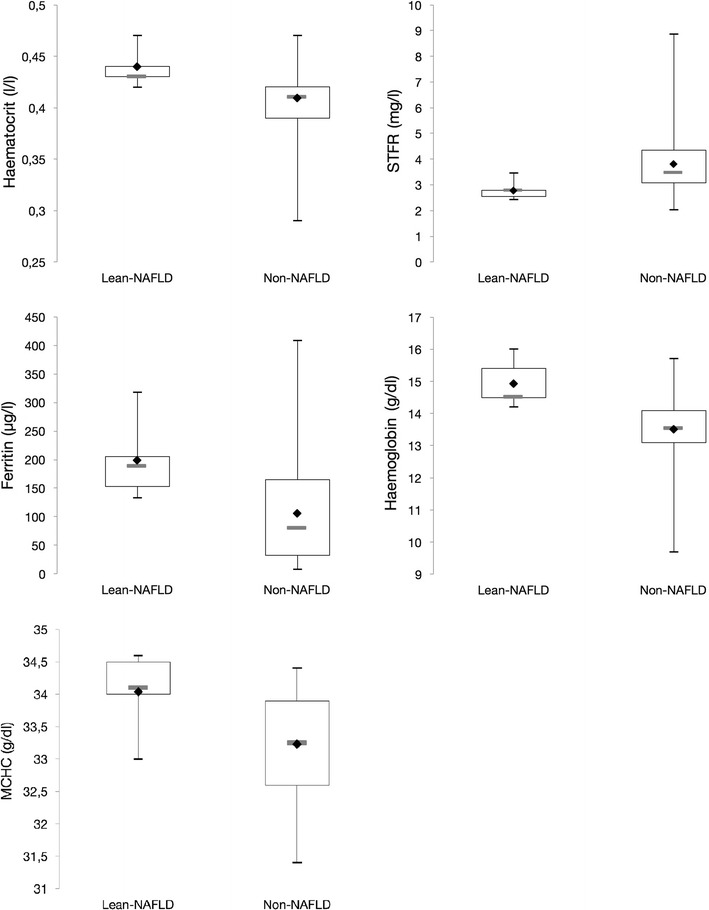
Box plots of haemoglobin/haematocrit/MCHC/ferritin and STFR values for the subject groups of lean NAFLD and non-NAFLD in the EMIL IIa study, Ulm University, 2013. EMIL: *Echinococcus multilocularis* and other medical conditions in Leutkirch; lean NAFLD = non-alcoholic fatty liver disease in slim people (BMI < 25); non-NAFLD = subjects with a high BMI (> 30) and no hepatic steatosis. Legend: upper whisker = maximum, lower whisker = minimum, rhombus = mean value of the data set, grey horizontal line in box = median, lower and upper end of box = lower (first, 25th percentile) and upper (third, 75th percentile) quartile

Values of serum ferritin, mean corpuscular haemoglobin concentration (MCHC), haemoglobin and haematocrit were significantly higher in the LN group, while soluble transferrin receptor was significantly lower than in the NN group (Fig. 2).

The HOMA-IR index was raised to an equal extent in the two groups, associated with mostly normal glycated haemoglobin (HbA1c) levels (below 6.5%) and an unremarkable proinsulin/insulin ratio (Additional files 1, 2).

Cholesterol and mean low-density lipoprotein (LDL) were raised in both groups, and the mean triglyceride concentration was above the upper limit of normal in the LN group (Fig. 2).

Analysis of the questionnaires showed an increased alcohol consumption in the LN group, although it was not above the exclusion criteria threshold (p = 0.003). There were no other diet-specific differences between the two groups (p > 0.05).

## Discussion

The prospective study presented here, comparing subjects with lean NAFLD and subjects with obesity (BMI > 30) but no demonstrable fatty liver infiltration, shows that iron metabolism parameters (haemoglobin, haematocrit, MCHC, serum ferritin, soluble transferrin receptor) are positively associated with a risk of lean NAFLD.

Recent research has shown that haemoglobin and haematocrit values correlate well with the HOMA-IR index or predominant insulin resistance in overweight patients [12]. Furthermore, it has been demonstrated that people with a raised haemoglobin level are at greater risk of developing abnormal liver function, and that the haemoglobin concentration in combination with triglycerides and serum ferritin levels may be a predictor of NAFLD [13].

Beaton et al. reported that ferritin levels are raised in people with NAFLD independently of inflammation [14]. Values associated with the red blood cells, and the possible effects on the haematopoietic system in the pathogenesis of lean NAFLD, have hardly been established or discussed in previous studies on lean NAFLD. One study on a Turkish patient population, published in 2014 by Akyuz et al., provided some evidence of raised haemoglobin levels in patients with lean NAFLD compared with overweight patients with NAFLD [15].

The current focus of research is the regulation of iron metabolism in individuals who have reduced hepcidin production or activity due to liver damage, e.g. with haemochromatosis [16, 17]. Liver cell damage in NAFLD may also lead to hepcidin deficiency and a low ferroportin breakdown rate; ferroportin hyperactivity leads to an increase in intestinal iron absorption and the uncontrolled release of iron from the reticuloendothelial system. Lu et al. demonstrated increased iron accumulation and the early development of liver fibrosis in hepcidin knockout mice [18]. Measurement of hepcidin and ferroportin in large-scale studies could reveal the relevance of these proteins as diagnostic markers.

An increased HOMA-IR index, as can also be seen in the results of the present study, indicates altered insulin sensitivity, not only in overweight subjects who do not have fatty liver changes, but also in those of normal weight with fatty liver disease. However, the question as to why insulin resistance does not lead to NAFLD in subjects with an increased BMI remains open.

HBA1c levels below 6.5% in at least 80% of the subjects in both groups show that advanced diabetes is not an issue.

Combining the blood parameters of serum ferritin and haemoglobin with the HOMA-IR index could possibly provide the basis for a clinical score to identify lean NAFLD, as has been discussed for NAFLD in obese patients [19].

Studies by Younossi et al. and Vos et al. in North-American or European populations showed clear differences in all hepatic transaminases, while our results did not [4, 20]. A study by Mofrad et al. demonstrated the possibility of NAFLD also developing when alanine aminotransferase (ALT) levels were within the normal range [21].

Mean values of both cholesterol and LDL were raised, emphasising the fact that a disorder of lipid metabolism can be present and may cause fatty liver disease in people of normal weight, but also that hyperlipidaemia need not necessarily lead to hepatic steatosis in obese subjects.

Anthropometric measurements did not provide any predictive results. The calculated WHR showed no significant difference between the groups and values that were overall normal or only slightly increased for women. Although the results of a study on a Chinese population showed a good prognostic value for the WHR, the direct extrapolation to a European population must be challenged [1].

Further efforts should be made to establish the terms “metabolically healthy but obese (MHO) individuals” and “metabolically obese but normal weight (MONW) individuals”, which have appeared in the medical literature for some years now [22]. A changed cardiovascular and metabolic profile has also been seen in a recent meta-analysis of 15 studies relating to lean and obese patients with NAFLD [23].

Moreover, the lack of hepatic steatosis in the overweight subjects demonstrates that there are protective factors, yet not researched, which can protect the individual from developing NAFLD, even when the blood lipids are raised and insulin resistance is increased. Examples include haemoxygenase-1, an enzyme involved in haem breakdown, which in one study was shown to have a catalytic effect on NAFLD and may be protective at low concentrations [24], and the identification and listing of intestinal bacteria in individuals with and without NAFLD [25].

Clarifying the specific genetic polymorphisms [especially PNPLA3 risk alleles (Patatin-like phospholipase domain-containing protein 3)] associated with a lean NAFLD, as shown in the study of Argo et al., and elucidating the phenotype responsible for the risk of developing the disease is one additional step towards improving the prevention und prognosis of the condition [26].

## Limitations

- Haemoglobinopathies such as thalassaemia, polycythaemia vera, and haemochromatosis, which are potential confounders or causes of iron overload, were not specifically excluded.
- Nor did we enquire about obstructive sleep apnoea as a cause of polycythaemia [27].
- Not all subjects complied with the 12-h fast, which may have partly affected the measured fasting blood glucose and ultrasound values. All subjects fasted for at least 4 h.
- Lack of liver-biopsies. We used ultrasound scans to make the diagnosis. Many studies addressing the same questions have also used ultrasound because it can be carried out more easily and has been shown to have a good specificity and sensitivity in the identification of moderate to severe fatty liver disease [28].
- Reduced number of subjects in the lean NAFLD group (5). Applying exclusion criteria meant a considerable reduction in the size of the groups. The specification and selection of the subjects from the 2002 EMIL I study meant that it was not possible to recruit more subjects. Our results can therefore be viewed as a basis for prospective clinical studies with larger numbers of subjects.
- We have to emphasise that the present study makes no attempt to demonstrate causality.

## Additional files

**Additional file 1: Table S1.** Gender and age distribution in the study population of the 2013 EMIL IIa study.

**Additional file 2: Table S2.** Previous studies on lean NAFLD with numbers of subjects/patients, lean NAFLD subjects/patients, and authors.

## Abbreviations

ALT: alanine transaminase
AntiTPO: antithyroid autoantibodies
AP: alkaline phophatase
AST: aspartate transaminase
BMI: body-mass-index
cm: centimetre
dl: decilitre
EDTA: ethylenediaminetetraacetic acid
EMIL: *Echinococcus multilocularis* and internal diseases in Leutkirch
fl: femtolitre
g: gram
GGT: Gamma-glutamyltransferase
HbA1c: glycated haemoglobin
HCT/Hk: haematocrit
HDL: high-density lipoprotein
HGB/Hb: haemoglobin
HOMA-IR: homeostasis model assessment-insulin resistance
kg: kilogram
l: litre
LDL: low-densitiy lipoprotein
MCH: mean corpuscular haemoglobin
MCHC: mean corpuscular haemoglobin concentration
MCV: mean corpuscular volume
Mg: milligram
MHO: metabollically healthy but obese
MHz: megahertz
ml: millilitre
mmol: millimol
MONW: metabolically obese but normal weight
MPV: mean platetet volume
µg: microgram
µl: microlitre
NAFLD: non-alcoholic-fatty-liver-disease
nmol: nanomol
OR: odds ratio
p: p-value
PNPLA3: patatin-like phospholipase domain-containing protein 3
pg: picogram
pmol: picomol
SD: standard deviation
SHBG: sex hormone-binding globulin
STFR: soluble transferrin receptor
T3: triiodothyronine
T4: thyroxine
TSH: thyroid-stimulating hormone
U: units
WHO: world health organisation
WHR: waist-to-hip-ratio
<: less-than
≤: less-than or equal to
>: greater-than
≥: greater-than or equal to
