# Supplementary material for: Laboratory parameters in lean NAFLD: comparison of subjects with lean NAFLD with obese subjects without hepatic steatosis
Source: BMC Res Notes. 2018 Feb 6;11:101. doi: 10.1186/s13104-018-3212-1 (PMC5801753; doi:10.1186/s13104-018-3212-1)
Supplement: Supplementary file 1 — Additional file 1: Table S1. Gender and age distribution in the study population of the 2013 EMIL IIa study. [file 13104_2018_3212_MOESM1_ESM.doc]

**Table 2 (separate additional files for online publication)**

***Table S1*** *Gender and age distribution in the study population of the 2013 EMIL IIa study. Comparison of the lean NAFLD (BMI<25 + fatty liver) and non-NAFLD (BMI>30 and no fatty liver) groups [EMIL= Echinococcus multilocularis and other medical conditions in Leutkirch]*

|  | **LEAN NAFLD**  **(LN)** | **NON-NAFLD  with obesity (NN)** |
| --- | --- | --- |
| **Gender n (%)**  **female**  **male** | 1 (20%)  4 (80%) | 21 (77.8%)  6 (22.2%) |
| **Age (years)**  **Mean ± SD, range** | 61.0 ± 10.1  46 - 71 | 56.1 ± 13.7  28 - 75 |
