# Supplementary material for: Laboratory parameters in lean NAFLD: comparison of subjects with lean NAFLD with obese subjects without hepatic steatosis
Source: BMC Res Notes. 2018 Feb 6;11:101. doi: 10.1186/s13104-018-3212-1 (PMC5801753; doi:10.1186/s13104-018-3212-1)
Supplement: Supplementary file 2 — Additional file 2: Table S2. Previous studies on lean NAFLD with numbers of subjects/patients, lean NAFLD subjects/patients, and authors. [file 13104_2018_3212_MOESM2_ESM.doc]

**Table 3 (separate additional files for online publication)**

***Table S2*** Previous studies on lean NAFLD with numbers of subjects/patients, lean NAFLD subjects/patients, and authors

| **Year of publication** | **Country** | **Number of subjects/patients** | **With lean NAFLD** | **Published by** |
| --- | --- | --- | --- | --- |
| **2004** | South Korea | 768 | 74 | Kim et al. |
| **2004** | South Korea | 360 | 120 | Park et al. |
| **2006** | Taiwan | 3245 | 61 | Chen et al. |
| **2007** | Singapore | 12 | 2 | Chow et al. |
| **2008** | South Korea | 408 | unclear | Jun et al. |
| **2009** | Japan | 56 | 12 | Yasutake et al. |
| **2010** | India | 1911 | 123 | Das et al. |
| **2011** | Belgium | 1777 | 31 | Vos et al. |
| **2012** | South Korea | 5878 | 1611 | Sinn et al. |
| **2012** | South Korea | 29994 | 3014 | Kwon et al. |
| **2012** | South Korea | 1161 | 107 | Chon et al. |
| **2012** | USA | 11613 | 431 | Younossi et al. |
| **2013** | India | 150 | 30 | Bhat et al. |
| **2013** | India | 541 | 48 | Vendhan et al. |
| **2013** | India | 336 | 27 | Kumar et al. |
| **2014** | Bangladesh | 465 | 119 | Alam et al. |
| **2014** | China | 1779 | 731 | Feng et al. |
| **2015** | Turkey | 483 | 37 | Akyuz et al. |
| **2016** | Austria | 187 | 55 | Feldmann et al. |
